# Lack of H5N1 Avian Influenza Transmission to Hospital Employees, Hanoi, 2004

**DOI:** 10.3201/eid1102.041075

**Published:** 2005-02

**Authors:** Nguyen Thanh Liem, World Health Organization International Avian Influenza Investigation Team, Vietnam, Wilina Lim

**Affiliations:** * National Pediatric Hospital, Hanoi, Vietnam;; † Department of Health, Hong Kong, Special Administrative Region, China

**Keywords:** Avian influenza A virus, patient- to-professional disease transmission, seroepidemiologic study

## Abstract

To establish whether human-to-human transmission of influenza A H5N1 occurred in the healthcare setting in Vietnam, we conducted a cross-sectional seroprevalence survey among hospital employees exposed to 4 confirmed and 1 probable H5N1 case-patients or their clinical specimens. Eighty-three (95.4%) of 87 eligible employees completed a questionnaire and provided a serum sample, which was tested for antibodies to influenza A H5N1. Ninety-five percent reported exposure to >1 H5N1 case-patients; 59 (72.0%) reported symptoms, and 2 (2.4%) fulfilled the definition for a possible H5N1 secondary case-patient. No study participants had detectable antibodies to influenza A H5N1. The data suggest that the H5N1 viruses responsible for human cases in Vietnam in January 2004 are not readily transmitted from person to person. However, influenza viruses are genetically variable, and transmissibility is difficult to predict. Therefore, persons providing care for H5N1 patients should continue to take measures to protect themselves.

## Abstract 2

A seroprevalence study found no transmission of avian influenza H5N1 viruses from patients to hospital employees in Vietnam, 2004.

Direct transmission of H5N1 viruses of purely avian origin from birds to humans was first described during an outbreak among poultry in Hong Kong in 1997. In that outbreak, 6 of 18 confirmed human H5N1 case-patients died (*1*), and serologic evidence was found for asymptomatic infection in humans after exposure to infected poultry (*2*).Avian-to-human transmission of influenza viruses is believed to be infrequent because of host barriers to infection, such as cell receptor specificities, and because the acquisition by avian viruses of the ability for human-to-human transmission requires either genetic reassortment with a human influenza strain or genetic mutation (*3*). However, a study of household and social contacts of Hong Kong H5N1 case-patients found evidence, although limited, for human-to-human transmission (*4*). Further evidence was provided by a study of healthcare workers (HCWs), which found that significantly more HCWs exposed to patients with H5N1 infection were positive for H5 antibody than nonexposed HCWs (3.7% vs. 0.7%); 2 HCWs seroconverted after exposure to H5N1-infected patients, in the absence of known poultry exposure (*5*). These 2 studies provided the first evidence, although limited, of human-to-human transmission of H5N1 viruses of purely avian origin.

On December 12, 2003, influenza A H5N1 viruses were detected among poultry at a farm near Seoul, the Republic of Korea (*6*), and outbreaks of H5N1 in poultry were subsequently reported in 8 other Asian countries (Japan, Indonesia, Vietnam, Thailand, Laos, Cambodia, China, and Malaysia); a situation that the Office International des Epizooties has called "a crisis of global importance" (*7*). Human case-patients infected with H5N1 related to these poultry outbreaks were identified in Vietnam and Thailand in January 2004, and on September 28, 2004, possible human-to-human transmission was reported in a family cluster in Thailand (*8*).

Concern is widespread that the current situation in Asia favors the emergence of a highly pathogenic influenza virus with the ability for efficient transmission from person to person, which would lead to an influenza pandemic. While experiences from Hong Kong in 1997 indicate that human-to-human transmission of purely avian H5N1 viruses is possible but not sustainable, genetic alterations over time may lead to subsequent H5N1 infections behaving quite differently. An understanding of the current and absolute risk for human-to-human transmission of circulating avian H5N1 viruses is vital to guide appropriate public health and infection control responses and to inform pandemic preparedness. Unfortunately, little data are available to quantify the transmissibility of the H5N1 strains currently circulating in poultry in Asia. To investigate the risk for human-to-human transmission of avian H5N1 viruses to hospital employees, we undertook a cross-sectional seroprevalence study among employees of 1 hospital in Vietnam, who were exposed to confirmed and probable H5N1 case-patients or their clinical samples.

## Methods

From December 27, 2003, to January 19, 2004, 4 children, 4–12 years of age, with confirmed H5N1 infection and 1 with probable H5N1 infection were admitted and treated at the National Pediatric Hospital (NPH), Hanoi, Vietnam. Detailed information regarding the 4 confirmed H5N1 patients has been published elsewhere (*9*). Eligible study participants were hospital employees who had possible exposure to the patients with confirmed or probable H5N1 infections, such as by working in wards or entering rooms where H5N1 patients were admitted, or having handled clinical specimens from these patients. To allow sufficient time for seroconversion in any infected HCWs, the study took place 29 days after discharge of the last confirmed H5N1 patient. All eligible participants were provided with written and verbal information about the study and gave written consent for participation.

### Definitions

We used the following definitions in our study: study period, from date of admission of first confirmed case-patient (December 27, 2003) to 29 days after discharge of the last confirmed case-patient (February 17, 2004); confirmed H5N1 primary case patient, a patient admitted to NPH, Hanoi, from December 27, 2003, to January 19, 2004, inclusive with a respiratory illness and influenza A H5N1 virus detected in clinical specimens by either viral culture or reverse transcriptase–polymerase chain reaction; probable H5N1 primary case patient, a patient admitted to NPH, Hanoi, from December 27, 2003, to January 19, 2004, inclusive with a respiratory illness and high titer of antibodies to influenza A/H5 detected in a single serum sample; possible H5N1 secondary case, a hospital employee who had fever (if measured >38°C), and at least 1 of 3 symptoms (cough, shortness of breath, sore throat), and contact with a confirmed or probable influenza A H5N1 case-patient, in the absence of exposure to poultry.

### Questionnaires

Information was collected by using a self-administered questionnaire in Vietnamese. Participants were asked their age, sex, residence address, occupation, department where they worked, whether they smoked, their medical history, whether they had symptoms during the study period, whether they had taken hygienic measures while caring for H5N1 case-patients, their influenza vaccination status, use of oseltamivir prophylaxis, and potential risk factors for H5N1. These risk factors included duration and type of exposure to H5N1 case-patients, contact with ill poultry or poultry that died of an illness, and whether they shopped at live-poultry markets or had freshly butchered or live poultry in their home in the previous month.

### Serologic Testing

All participants were asked to provide a single blood specimen. Serum samples were collected on February 17, 2004, immediately processed, stored at –25°C, and shipped frozen on dry ice to the Government Virus Unit, Department of Health, Hong Kong, China. Serum samples were tested for antibodies to influenza A H5N1 virus by microneutralization test as described by Rowe et al. (*10*) with H5N1 viruses A/Vietnam/1194/2004 and A/Vietnam/3212/2004. Serum was considered to be positive in the microneutralization test if an anti-H5 titer of >40 was obtained in 2 independent assays. Microneutralization antibody-positive serum was adsorbed with influenza A H1N1 virus to eliminate the possibility of detecting antibody that was cross-reactive among influenza virus of different subtypes, and the microneutralization test was repeated. No change in antibody titer after adsorption indicated the presence of anti-H5 antibody, while a >4-fold reduction in microneutralization after adsortion was interpreted as evidence for significant cross-reaction. Microneutralization antibody-positive serum was subjected to Western blot analysis by using recombinant protein from A/HK/156/97 virus.

## Results

### Study Participants

Of 87 eligible staff members who had possible exposure to H5N1 patients, 83 (95.4%) completed a questionnaire and provided a serum sample (Table 1). The median age of employees was 37.4 years (range 22–55 years), and 53 (64%) were female. Most employees (97.6%) were residents of Hanoi City, Vietnam. Of the 83 employees, 51 (61%) were nurses or nurse's aides, 19 doctors (23%), 7 (8%) laboratory employees, and 6 (7%) other. Thirty-seven (45.1%) worked in the intensive care unit (ICU), 30 (36.6%) in the infectious diseases department, 8 (9.8%) in the laboratory, 6 (7.3%) in radiology, and 1 in the hematology department. More than two thirds (68.3%) of the employees reported receiving influenza vaccine in 2004, and 1 person reported taking oseltamivir for treatment of influenzalike illness since December 27, 2003. No respondents took oseltamivir as prophylaxis against influenza infection. In total, 76.8% of participants reported contact with 2 or 3 influenza A H5N1 patients. Four hospital employees (4.9%) reported no contact with H5N1 patients; they were all laboratory personnel who had handled clinical material from H5N1 patients. Median duration of exposure to the hospitalized H5N1 primary case-patients reported was 82 hours, ranging from 1 to 299 hours (N = 78). Most participants reported always wearing protective masks (94.8%), gloves (61.5%), and eye-protection (31.6%) while caring for H5N1 patients (Table 2).

**Table 1:** Demographic and exposure characteristics of study participants

Characteristic	n (%)*
Age group (y)	
<30	20 (24.1)
30–39	26 (31.3)
40–49	26 (31.3)
>49	11 (13.3)
Male sex	30 (36.1)
Residence in Hanoi City	81 (97.6)
Department	
ICU†	37 (45.1)
Infectious diseases	30 (36.6)
Laboratory	8 (9.8)
Radiology	6 (7.3)
Hematology	1 (1.2)
Years smoked	
None	64 (78.1)
<10	6 (7.3)
10–30	12 (14.6)
Chronic medical condition	22 (26.5)
Influenza vaccination in 2004	56 (68.3)
Oseltamivir since Dec 27, 2003	1 (1.2)
No. of H5N1 patients visited	
0 (none)	4 (4.9)
1	3 (3.7)
2	31 (37.8)
3	32 (39.0)
4	4 (4.9)
5 (all)	8 (9.8)
Changed bedding	
Yes	46 (59.0)
No	32 (41.0)
Touched patients	
Yes	75 (96.2)
No	3 (3.9)
Exposure to H5N1 patient(s) who did not wear masks	57 (73.1)


*The no. of study participants (n) for each characteristic ranged from 78 to 83; percentage provided is based on the actual number of participants. †ICU, intensive care unit.

**Table 2:** Protective equipment used by hospital employees while examining or caring for H5N1 patients

Equipment	n (%)
Mask (N = 77)	
Always	73 (94.8)
Not always	2 (2.6)
Never	2 (2.6)
Types of masks (N = 75)*	
N95	65 (86.7)
Surgical	55 (73.3)
N92	2 (2.7
Other	8 (10.7)
Eye protection (N = 76)	
Always	24 (31.6)
Not always	15 (19.7)
Never	37 (55.2)
Type of eye protection (N = 39)	
Glasses	36 (92.3)
Face shield	3 (7.7)
Gloves (N = 78)	
Always	48 (61.5)
Not always	21 (26.9
Never	9 (11.5)

*Use of multiple respirators or masks at different times possible.

### Clinical Symptoms

The figure summarizes the symptoms reported by hospital employees during the study period. Overall, 59 (72.0%) employees reported symptoms during the study period; 66.0% of these had onset of symptoms within 1 to 7 days after exposure to a H5N1 patient. Median duration of reported illness was 5 days (range 0–40 days). Three persons (5.4%) were too ill to work; none were admitted to the hospital. Two persons (2.4%) who worked in ICU met the possible secondary H5N1 case-patient definition. They reported contact with patients but not with sick poultry or pigs, and neither worked in the laboratory. Both reported receiving the 2003–2004 influenza vaccine and denied taking oseltamivir. Table 3 summarizes reported contact with poultry and pigs by participants. Approximately 1 quarter of participants (25.6%) reported the presence of poultry outside their homes, and 2 HCWs (9.5%) reported that poultry had died in the past month. The 2 possible H5N1 secondary case-patients did not report have poultry dying outside their homes within the previous month.

**Figure:**
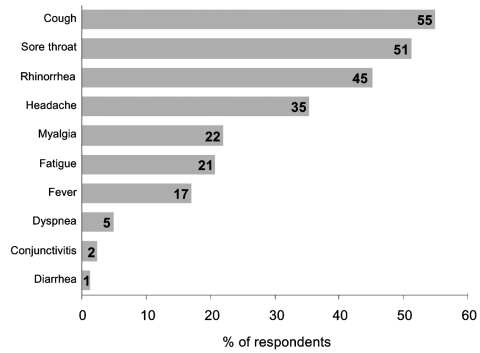
Figure. Reported symptoms and percentage of hospital employees with symptoms (N = 82).

**Table 3:** Possible non–healthcare-related H5N1 exposures among study participants

Exposure	n (%)*
Poultry outside the home in last 4 weeks	21† (25.6)
Do not know	6 (7.3)
Pigs outside the home in the last 4 weeks	10‡ (12.2)
Do not know	7 (8.5)
Visited market with sick poultry in last 4 weeks	3 (3.7)
Do not know	18 (22.2)
<1 m from sick or dead poultry since July 2003	8 (10.)
Do not know	11 (13.6)
Anyone sick in the household in the last week	11 (13.4)
Do not know	2 (2.4)

*The no. (N) of study participants for each characteristic was 80 to 82 with the percentage provided based on the actual number of respondents. †Of which 2 persons had dying poultry outside their home. ‡Of which none had dying pigs outside their home.

### H5N1 Antibody Prevalence

Samples were obtained from all 83 participants, including the 2 with possible secondary cases, and none were positive for antibodies to influenza A H5N1. One sample initially had an antibody titer of 160 and 640 against A/Vietnam/1194/2004 and A/Vietnam/3212/2004, respectively. However, microneutralization tests using influenza A H1N1 viruses showed a high titer of 10,240, and microneutralization repeated after adsorption with influenza A H1N1 virus showed an 8-fold reduction in the antibody titer, which was interpreted as indicating a cross-reacting anti-N1 antibody.

## Discussion

No evidence was found of nosocomial transmission of H5N1 viruses among 83 hospital employees with exposure to 4 confirmed and 1 probable H5N1 case-patients or their clinical samples. A number of possible factors may explain these findings: a lack of infectivity of the patients at the time of admission; the effective use of personal protective equipment (PPE) and infection control; low sensitivity of the antibody detection method; lack of susceptibility of HCWs, or a lack of transmissibility of this particular H5N1 strain.

No data are available on the duration of H5N1 virus shedding in children. However, for human influenza virus, viral shedding at high titers is generally more prolonged in children, and virus can be recovered up to 6 days before and 21 days after the onset of symptoms. The H5N1 patients in this study were admitted with severe illness 3–7 days after onset of symptoms and PCR-positive specimens were obtained from the 4 confirmed case-patients on the day 1 (1 patient), day 2 (1 patient), and day 3 (2 patients) after admission. In addition, live virus was cultured from samples taken from 2 of the patients on days l and 3 after admission, respectively. None of the patients were treated with oseltamivir because this was not available at the time (*9*). Two of the patients were treated orally with the nucleoside analogue ribavirin during their admission, 1 on day 4 after admission, and the other on day 1 (*9*). However, the 2 other confirmed case-patients and the probable case-patient did not receive antiviral treatment and, if human infection with H5N1 is associated with viral shedding, these patients would be expected to be contagious during their admission.

Most hospital employees (94.8%) reported that they always wore masks while caring for H5N1 patients, and often the reported type of mask was an N95 respirator. However, N95 respirators were first available in NPH on January 7, and some employees reported wearing N95s before this date. Therefore, reported PPE use in this study may be biased by inaccurate recall or a tendency to report behavior that HCWs know is recommended. Enhanced infection control practices and PPE were instituted on January 7, and the diagnosis of avian influenza was first confirmed on January 9. Therefore some HCWs in this study were likely exposed to H5N1 patients without optimal PPE or infection control.

Oseltamivir prophylaxis was not used by any of the staff in this study and therefore did not play a role in protecting HCWs. Whether the HCWs in the study were protected by cross-reactive immunity to other influenza A subtypes is hard to assess. One possible explanation for the observation that most confirmed H5N1 case-patients are reported in children or young adults is that older adults are protected by cross-reactive immunity from previous exposure to other influenza A viruses. This hypothesis requires further investigation.

Serum samples were taken from HCWs at least 29 days after last possible exposure and at a time when the antibody response to exposure would be expected to be detectable (*4*). Based on a small number of samples, the sensitivity of microneutralization test in detecting antibodies to H5N1 in children and adults is 88% and 80%, respectively, while the specificity is 100% and 93%, respectively (*10*). Also, the microneutralization assay utilized H5N1 strains isolated from human patients in North Vietnam, so the negative results are unlikely to be false negatives due to a poor match between antigen and antibody. False-positive results are perhaps more likely, and 1 sample was initially positive but appeared to be due to cross-reacting anti-N1 antibody.

Epidemiologic evidence from Vietnam and Thailand clearly indicates that sustained human-to-human transmission of H5N1 has not yet occurred. Most reports of H5N1-infected patients have been sporadic, and despite the evidence from Hong Kong of human-to-human transmission and the occurrence of family clusters of H5N1 in Vietnam and Thailand, no evidence indicates that influenza A H5N1 has ever caused >1 generation of human-to-human transmission. Although this study has not distinguished the inherent transmissibility of the virus from the influence of infection control or host resistance, the data provides further reassurance that the risk for human-to-human transmission of currently circulating avian H5N1 viruses is low. Studies among household members of confirmed H5N1 case-patients will provide additional information on the risk for human-to-human transmission in the absence of infection control measures.

While the absolute risk for human-to-human transmission of avian H5N1 viruses may be low at this time, the high case-fatality proportion seen among recent human H5N1 patients demonstrates that the individual consequences of infection are very serious and intensive measures to protect healthcare workers and laboratory staff against infection remain warranted. The risk of human-to-human transmission of H5N1 viruses could increase in the future. Consequently, every H5N1 case should be managed by clinicians and public health professionals with the assumption that human-to-human transmission can occur and that the risk for such transmission is unpredictable.

## References

[1] Yuen KY, Chan PK, Peiris M, Tsang DNC, Que TL, Shortridge KF, Clinical features and rapid viral diagnosis of human disease associated with avian influenza A H5N1 virus. Lancet. 1998;351:467–71. 10.1016/S0140-6736(98)01182-9 9482437

[2] Buxton Bridges C, Lim W, Hu-Primmer J, Sims L, Fukuda K, Mak KH, Risk of influenza A (H5N1) infection among poultry workers, Hong Kong, 1997–1998. J Infect Dis. 2002;185:1005–10. Epub Mar 19, 2002. 10.1086/340044 11930308

[3] Webster RG. Influenza virus: transmission between species and relevance to emergence of the next human pandemic. Arch Virol Suppl. 1997;120(Suppl 120):105–13. 9413531 10.1007/978-3-7091-6534-8_11

[4] Katz JM, Lim W, Bridges CB, Rowe T, Hu-Primmer J, Lu X, Antibody response in individuals infected with avian influenza A (H5N1) viruses and detection of anti-H5 antibody among household and social contacts. J Infect Dis. 1999;180:1763–70. 10.1086/315137 10558929

[5] Buxton Bridges C, Katz JM, Seto WH, Chan PK, Tsang D, Ho W, Risk of influenza A (H5N1) infection among health care workers exposed to patients with influenza A (H5N1), Hong Kong. J Infect Dis. 2000;181:344–8. 10.1086/315213 10608786

[6] Office International des Epizooties. Disease information bulletin. 12 December 2003. Vol. 16 - No. 50. [accessed 2004 October 11]. Available from http://www.oie.int/eng/info/hebdo/AIS_67.HTM#Sec2

[7] Office International des Epizooties. Press release. Update on highly pathogenic avian influenza control methods in Asia including use of vaccination [accessed 2004 October 11]. Available from http://www.oie.int/eng/press/en_040927.htm

[8] Thailand Ministry of Public Health. Press Release: Avian influenza infectious of patients in Kamphaeng-Phet (Sept 28, 2004) [accessed 2004 October 11]. Available from http://thaigcd.ddc.moph.go.th/download/AI_press_280904_en.pdf

[9] Hien TT, Liem NT, Dung NT, San LT, Mai PP, Chau NvV, Avian influenza A (H5N1) in 10 patients in Vietnam. N Engl J Med. 2004;350:1179–88. 10.1056/NEJMoa040419 14985470

[10] Rowe T, Abernathy RA, Hu-Primmer J, Thompson WW, Lu X, Lim W, Detection of human serum antibody to avian influenza A (H5N1) virus using a combination of serologic assays. J Clin Microbiol. 1999;37:937–43. 10074505 10.1128/jcm.37.4.937-943.1999 PMC88628

